# Apatinib plus vinorelbine versus vinorelbine for metastatic triple-negative breast cancer who failed first/second-line treatment: the NAN trial

**DOI:** 10.1038/s41523-022-00462-6

**Published:** 2022-09-20

**Authors:** Dou-Dou Li, Zhong-hua Tao, Bi-Yun Wang, Lei-Ping Wang, Jun Cao, Xi-Chun Hu, Jian Zhang

**Affiliations:** Department of Medical Oncology; Fudan University Shanghai Cancer Center; Department of Oncology, Shanghai Medical College, Fudan University, Shanghai, China

**Keywords:** Breast cancer, Tumour angiogenesis

## Abstract

While therapies such as chemotherapy combined with immunotherapy, sacituzumab govitecan, and PARP inhibitors are available for metastatic TNBC, on disease progression after these therapies, the mainstay of therapy is chemotherapy. Apatinib is a small-molecule tyrosine kinase inhibitor that has promising anti-angiogenesis and antitumor activity for TNBC. We aimed to evaluate the safety and efficacy of adding apatinib to chemotherapy in patients with advanced TNBC with failed first/second-line treatment. A total of 66 patients were randomly assigned, in a 1:1 ratio, to receive vinorelbine or vinorelbine with apatinib in 28-day cycles. The primary endpoint was progression-free survival (PFS). Secondary endpoints included overall survival (OS), overall response rate (ORR) and safety. 33 received apatinib plus vinorelbine and 32 received vinorelbine (1 was withdrawal). Median PFS was significantly longer in the apatinib plus vinorelbine group than in the vinorelbine group (3.9 months vs. 2.0 months; hazard ratio, 1.82; 95% confidence interval [CI], 1.06 to 3.11; *P* = 0.026). Median OS was 11.5 months with apatinib plus vinorelbine and 9.9 months with vinorelbine (HR,1.01; 95% CI, 0.51 to 1.97; *P* = 0.985). The ORR was 9.1% in the apatinib plus vinorelbine group and 6.3% in the vinorelbine group (*P* = 0.667). The most common treatment-related hematologic grade 3–4 adverse events in apatinib plus vinorelbine group, were leukopenia, granulocytopenia, anemia, and thrombocytopenia. no treatment-related nonhematologic grade 4 adverse events or treatment-related deaths were observed. Collectively, adding apatinib to vinorelbine shows a promising benefit in PFS compared to vinorelbine monotherapy, with an excellent toxicity profile, warranting further exploration.

## Introduction

Breast cancer is the most frequently diagnosed malignancy among female worldwide and is a highly heterogeneous disease with diverse molecular profiles, which is closely related to prognosis and treatment response^1,2^. Triple-negative breast cancer (TNBC) accounts for 12–20% of all invasive breast cancer cases. It is characterized by negative estrogen receptor (ER), progesterone receptor (PR), and nonamplified human epidermal growth factor receptor 2 (HER2)^3,4^. Compared with other subtypes, TNBC is characterized by high histological grade, early onset (<50 years), malignancy aggressiveness, strong invasion, high risk of postoperative recurrence and metastasis, high probability of visceral and brain metastasis, rapid disease progression, and poor clinical prognosis^5^.

Chemotherapy remains the mainstay of treatment, which is limited by short duration of response and considerable toxicity. Although the combination of chemotherapy and immunotherapy or PARP inhibitors prolonged the survival to a certain extent, but the results for patients with metastatic TNBC remain significantly poor compared to other subtypes^6–10^. No standard treatment exists for TNBC with failure of multi-line therapies. there is no clear favored sequencing of agents after the first few lines—there are several standard therapies for metastatic TNBC. Novel treatment approaches that target this population of patients are desperately needed.

Anti-angiogenic drugs have been gradually attempted in the treatment of TNBC due to its higher expression of VEGF and VEGFR compared with other subtypes of breast cancer, despite the side effects of bleeding, hypertension, and thrombus^11–13^. Since 2009, several phase III trials have investigated anti-angiogenesis molecular targeted therapies in metastatic TNBC^14–16^. Randomized clinical trials in metastatic breast cancer document that the addition of bevacizumab to chemotherapy agents modestly improves time to progression and response rates^17–19^. The time to progression impact may vary among cytotoxic agents and appears greatest with bevacizumab in combination with weekly paclitaxel^20^. Besides, none of these studies demonstrates an increase in OS or Quality of life (QOL) when analyzed alone or in a meta-analysis of the trails^21^. Although bevacizumab does not benefit PFS conversion to OS or QOL, anti-angiogenic drugs remain an important part of the treatment of metastatic breast cancer. The attempt of small molecule anti-angiogenesis inhibitors is also worthy of further exploration. Apatinib is a novel small-molecule tyrosine kinase inhibitor targeting vascular endothelial growth factor receptor-2, which opens a new era of oral targeted anti-angiogenesis therapy^22–25^. In our previous clinical study NCT01176669, we evaluated the optimum dose level for the efficacy and safety of apatinib monotherapy in heavily pretreated patients with metastatic TNBC in China. The results showed that apatinib (at a dose of 500 mg once daily) showed promising efficacy and safety in salvage treatment of TNBC. The median PFS was 3.3 months (95% CI, 2.2–4.5), and the median OS was 11.1 months (95% CI, 5. 1–17. 2), which was superior to sunitinib^26^.

With the increasing use of anthracyclines and taxanes in adjuvant and neoadjuvant treatment of early breast cancer, the range of effective chemotherapy drugs for advanced TNBC is further limited^27^. A considerable number of patients have received capecitabine or cisplatin during first few lines. Thus, vinorelbine is one of the few posterior line drugs that can be selected. Certain efficacy of vinorelbine had been observed in previous clinical practices, which can be set as the control group (25 mg/m^2^, intravenous drip, 1, 8, 15 days)^28^. According to the viewpoint of tumor vascular normalization: there exists a time window for normalizing the structure and function of tumor blood vessels after anti-angiogenic therapy, during which the sensitivity of tumor to chemoradiotherapy increases^29,30^. Combined with our previous clinical trials NCT01176669, we confirmed the efficacy and safety of apatinib monotherapy in heavily pretreated patients with metastatic TNBC in our center. However, there is no published literature on chemotherapy combined with apatinib.

The NAN trial, a prospective, open label, phase II multicenter trial, was designed to further investigate whether apatinib administration before chemotherapy (apatinib 250 mg oral, qd, days 1–5, 8–12, 15–19) can increase the efficacy of vinorelbine by taking advantage of the time window of vascular normalization in women with advanced TNBC.

## Result

### Patients

**Fig. 1:**
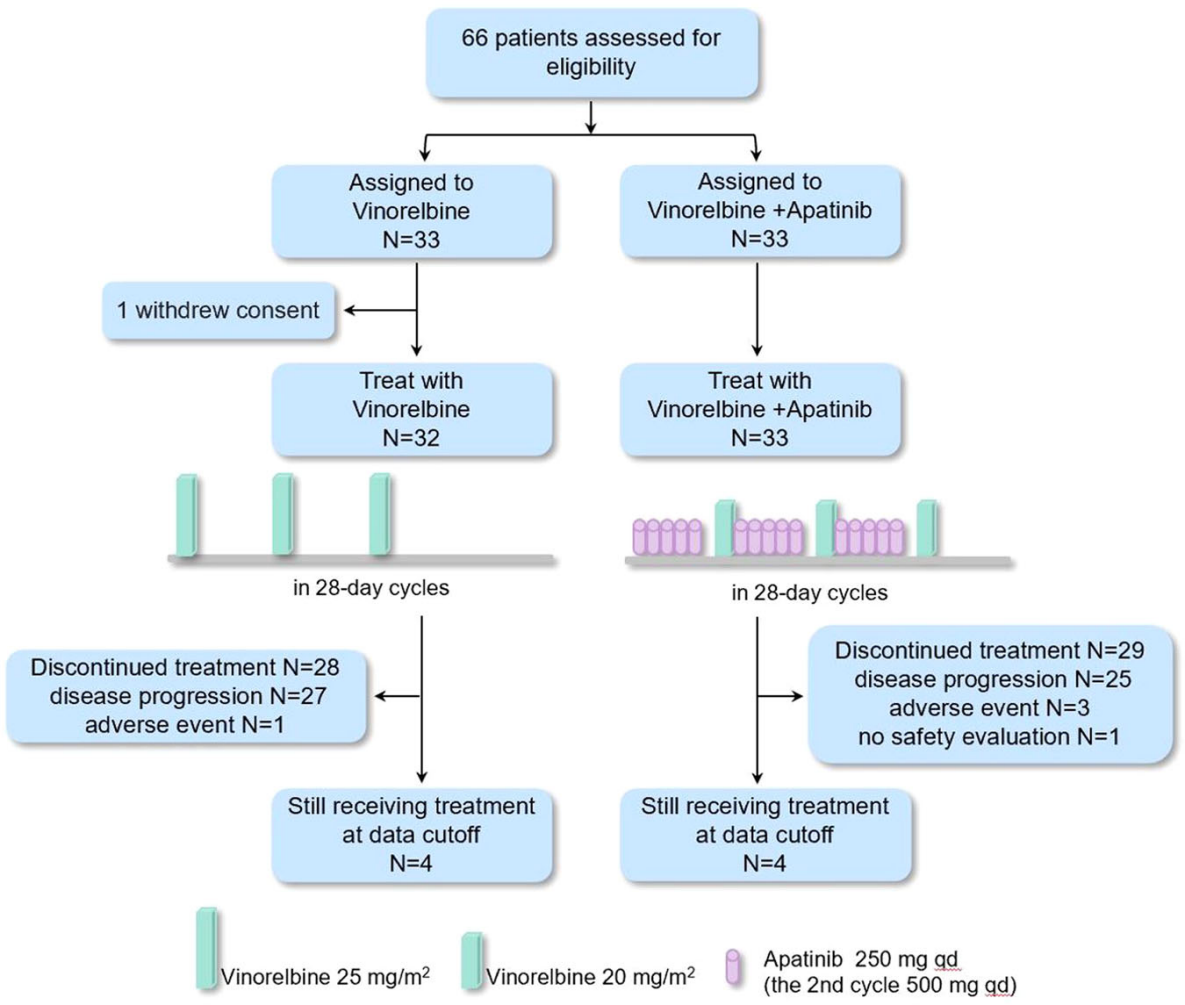
Enrollment, randomization, and treatment. A total of 66 patients underwent randomization. 33 received apatinib plus vinorelbine treatment, 32 patients who were randomly assigned to vinorelbine monotherapy and 1 patient withdrew consent without receiving treatment. Vinorelbine monotherapy was administered as a once-per-week intravenous infusion of 25 mg/m^2^ on days 1, 8, and 15 of each 28-day treatment cycle. In vinorelbine plus apatinib group, vinorelbine in administered intravenously at a dose of 20 mg/m^2^ on day 7, 14, and 21 of each 28-day treatment cycle. Apatinib 250 mg was administered orally once daily at a fixed time each day about 30 min after a meal, with treatment on days 1 to 5 of weeks 1, 2, and 3 within each 28-day cycle (if tolerable, the second cycle started with 500 mg per day).

Between September 14, 2017 and December 08, 2020, a total of 66 patients underwent randomization. 33 received apatinib plus vinorelbine treatment, 32 patients who were randomly assigned to vinorelbine monotherapy and 1 patient withdrew consent without receiving treatment (Fig. 1). Demographic distributions and clinicopathologic characteristics of the enrolled patients are summarized in Table 1. The mean age of included patients at the time of diagnosis was 48 years. All the patients were diagnosed with invasive ductal carcinoma with metastatic disease. Visceral metastatic rate in vinorelbine monotherapy group and apatinib plus vinorelbine group was 19% and 27%, respectively. Accordingly, 15% and 19% of patients had metastases in ≥3 organs. A majority of patients had received prior anthracycline or paclitaxel-based adjuvant or neoadjuvant chemotherapy and platinum-based metastatic chemotherapy. The median duration of follow-up was 22.3 months (range, 1.8–41.2). The data cutoff date was January 31, 2021.

**Table 1:** Baseline characteristics of the patients.

	Vinorelbine Group (*N* = 32)	Vinorelbine + Apatinib Group (*N* = 33)	
Characteristic	No. (%)	No. (%)	*P*
Age — yr			0.66
Median	48	48	
Range	31-68	25-70	
Age of enrollment			0.44
≤40	7(21.9)	10(30.3)	
>40	25(78.1)	23(69.7)	
ECOG performance status			0.61
0	21(65.6)	19(57.6)	
1	11(34.4)	14(42.4)	
Menopausal status			0.93
Premenopausal	10(31.3)	10(30.3)	
Postmenopausal	22(68.8)	23(69.7)	
Sites of metastatic disease			
Liver	6(18.8)	14(42.4)	0.04
Lung	16(50.0)	17(51.5)	0.90
Bone	9(28.1)	12(36.4)	0.48
Brain	0(0)	2(6.1)	0.49
Lymph nodes	25(78.1)	24(72.7)	0.61
No. of metastatic sites			0.88
≤3	17(53.1)	14(42.4)	
>3	15(46.9)	19(57.6)	
Visceral metastatic disease			0.05
Yes	19(59.4)	27(81.8)	
No	13(40.6)	6(18.2)	
Previous surgery			0.71
Yes	28(87.5)	30(90.9)	
No	4(12.5)	3(9.1)	
Previous radiotherapy			0.17
Yes	13(40.6)	19(57.6)	
No	19(59.4)	14(42.4)	
Previous chemotherapy			
Anthracycline	1(3.1)	1(3.0)	0.98
Paclitaxel	3(9.4)	2(6.1)	0.67
Anthracycline+ Paclitaxel	25(78.1)	29(87.9)	0.34
Previous platinum-based chemotherapy	32(100)	31(93.9)	0.49
Previous platinum adjuvant or neoadjuvant chemotherapy	2(6.3)	3(91)	0.67
Previous platinum metastatic chemotherapy	32(100)	31(93.9)	0.16

*ECOG* European Cooperative Oncology Group.

### Efficacy

**Fig. 2:**
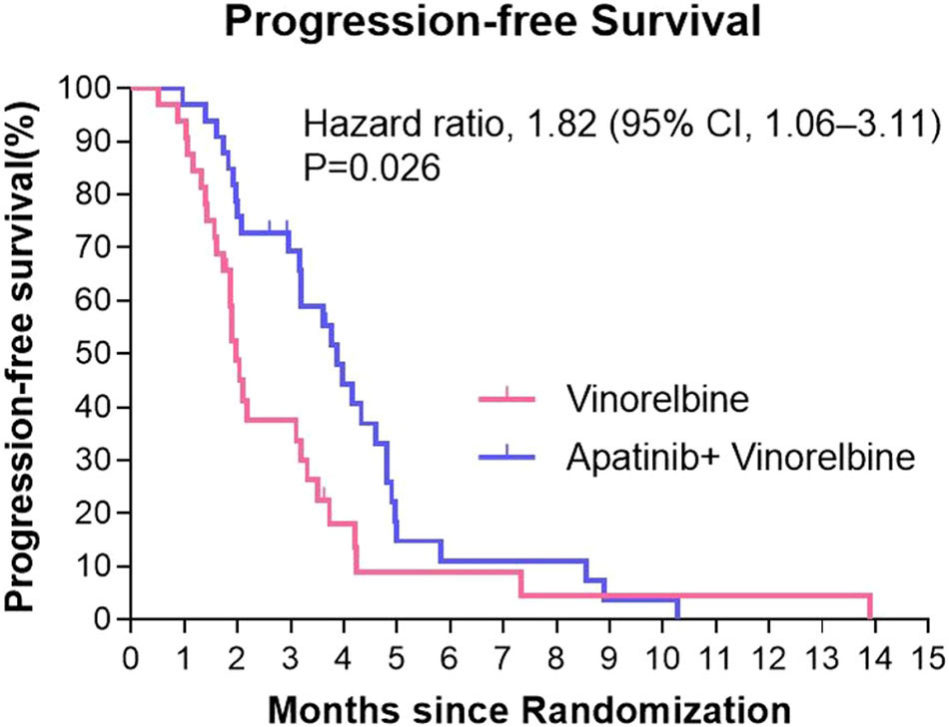
Kaplan–Meier Estimates of Progression-free Survival among patients in the vinorelbine group and apatinib plus vinorelbine group.

**Fig. 3:**
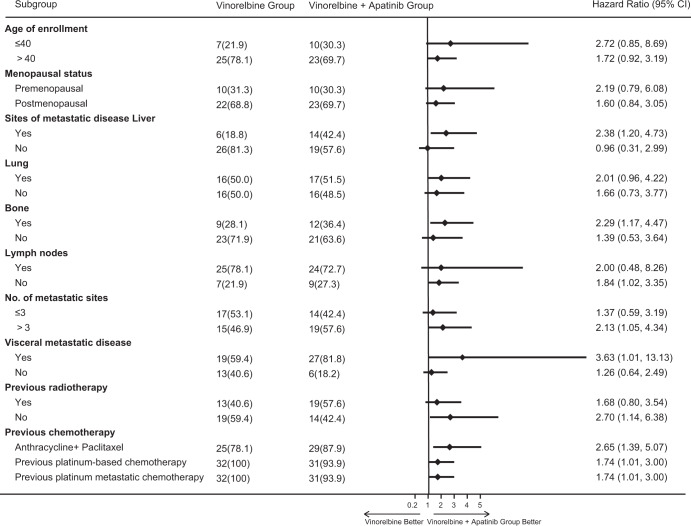
Subgroup Analysis of Progression-free Survival among patients in the vinorelbine group and apatinib plus vinorelbine group.

We conducted survival analysis to evaluate the two groups on PFS by Kaplan‐Meier method. As shown in Fig. 2, Median PFS was significantly longer in the apatinib plus vinorelbine group than in the vinorelbine group (3.9 months vs. 2.0 months; hazard ratio for disease progression or death, 1.82; 95% confidence interval [CI], 1.06–3.11; *P* = 0.026). A total of 12.1% of the patients in the apatinib plus vinorelbine group and 15.6% of the patients in the vinorelbine group and did not have disease progression at data cutoff. Subgroup analysis of progression-free survival in the vinorelbine group and the apatinib plus vinorelbine group is summarized in Fig. 3. After analyzing with clinically relevant factors including age, menopausal status, number of metastases, previous treatment, visceral metastasis, our data showed that the risk of disease progression in apatinib plus vinorelbine group was apparently lower than that of patients in vinorelbine group.

**Fig. 4:**
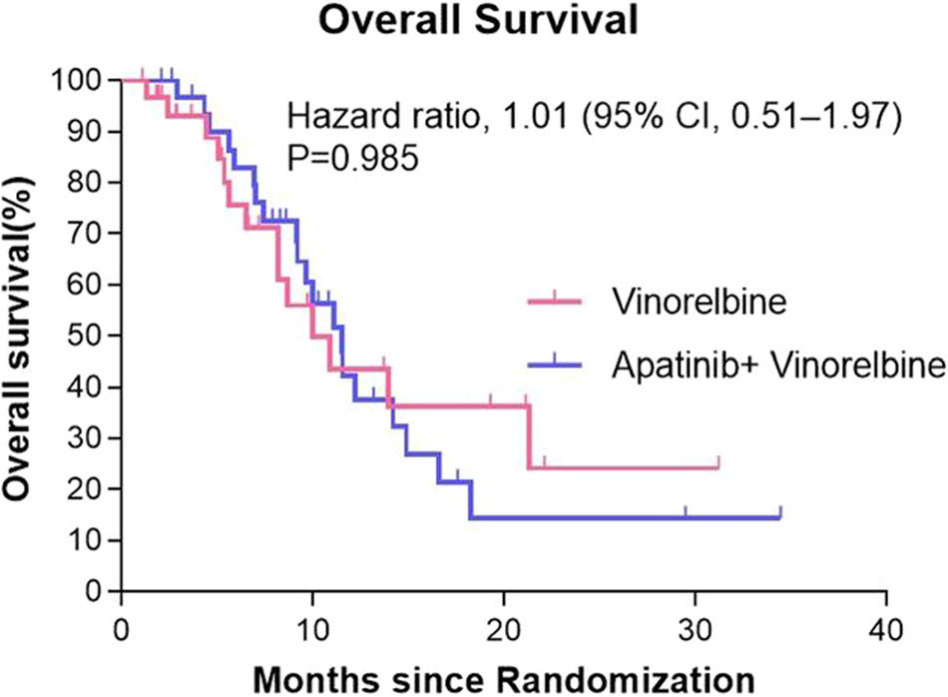
Kaplan–Meier Estimates of Overall Survival among patients in the in the vinorelbine group and apatinib plus vinorelbine group.

At the time of data cutoff, 34 patients had died (52.3%): 20 (60.6%) in the apatinib plus vinorelbine group and 14 (43.8%) in the vinorelbine group. Median OS was 11.5 months with apatinib plus vinorelbine and 9.9 months with vinorelbine (HR,1.01; 95% CI, 0.51 to 1.97; *P* = 0.985). (Fig. 4). According to RECIST 1.1, we investigated the objective response rate (ORR) between the two groups, and the results revealed that the ORR was 9.1% among patients who received apatinib plus vinorelbine and 6.3% among those who received vinorelbine monotherapy (*P* = 0.667). Two patients in each group achieved a partial response (PR). As compared with no patients in the vinorelbine group, one (3.0%) of 33 patients in apatinib plus vin orelbine group achieved a complete response (CR), with the maximum diameter of the target lesion reducing from 18.3 mm at baseline to 4.9 mm after 4 cycles of therapy, this patient maintained a CR for 8.6 months (Table 2). At the time of the last follow-up, there were one patient maintaining PR status in apatinib plus vinorelbine group.

**Table 2:** Clinical response to treatment.

Variable	Vinorelbine Group (*N* = 32)	Vinorelbine + Apatinib Group (*N* = 33)	*P*
Best overall response among patients with measurable disease— no. (%)			
Complete response	0(0.0)	1(3.0)	0.32
Partial response	2(6.3)	2(6.1)	0.98
Stable disease	9(28.1)	13(39.4)	0.34
Could not be evaluated	3(9.4)	3(9.1)	0.97
Objective response rate	2(6.3)	3(9.1)	0.67
Median (IQR) duration of treatment, months	1.6(0.3–12.7)	1.6(0.3–13.4)	

### Safety

All 65 patients received at least one cycle of apatinib plus vinorelbine or vinorelbine monotherapy and the median treatment duration was 1.6 months (range, 0.4 to 12.7) in the apatinib plus vinorelbine group and 1.4 months (range, 0.3–9.7) in the vinorelbine group. Three patients in each group were still receiving study treatment at the data cutoff date (January 2021). Adverse events of any grade are summarized in Table 3. The incidence of hematological toxicity was higher in the apatinib plus vinorelbine group, Grade 3 or 4 hematologic adverse events occurred in those treated with apatinib plus vinorelbine versus vinorelbine, respectively, were leukopenia (42.4% vs. 40.6%), granulocytopenia (57.6% vs. 31.3%), anemia (9.1% vs. 12.5%) and thrombocytopenia (3.0% vs. 3.1%). The most common nonhematologic toxicities were increased ALT (54.5% vs.37.5%), fatigue (27.3% vs.18.8%), nausea (18.2% vs.18.8%) and hand–foot syndrome (24.2% vs.15.6%), which occurred more frequently in apatinib plus vinorelbine group than vinorelbine group. the majority of nonhematologic adverse events in the vinorelbine group were grade 1–2 in severity.

**Table 3:** Summary of adverse events.

	Vinorelbine Group (*N* = 32)		Vinorelbine + Apatinib Group (*N* = 33)		*P*	
	Any Grade	Grade ≥ 3	Any Grade	Grade ≥ 3	Any Grade	Grade ≥ 3
Adverse event	No. (%)					
Hematologic						
Leukopenia	28(87.5)	13(40.6)	27(81.8)	14(42.4)	0.72	0.88
Neutropenia	24(75.0)	10(31.3)	29(87.9)	19(57.6)	0.18	0.03
Anemia	25(78.1)	4(12.5)	23(69.7)	3(9.1)	0.44	0.66
Thrombocytopenia	4(12.5)	1(3.1)	9(27.3)	1(3.0)	0.14	0.98
Nonhematologic						
Increased ALT/AST level	12(37.5)	0(0.0)	18(54.5)	1(3.0)	0.17	1.00
Increased Blood bilirubin	3(9.4)	0(0.0)	7(21.2)	0(0.0)	0.19	-
Anorexia	2(6.3)	0(0.0)	5(15.2)	0(0.0)	0.25	-
Nausea	6(18.8)	0(0.0)	6(18.2)	0(0.0)	0.95	-
Vomiting	2(6.3)	0(0.0)	2(6.1)	0(0.0)	0.98	-
constipation	3(9.4)	0(0.0)	2(6.1)	0(0.0)	0.62	-
Mucosal inflammation	1(3.1)	0(0.0)	0(0.0)	0(0.0)	0.31	-
Hand–foot syndrome	5(15.6)	0(0.0)	8(24.2)	1(3.0)	0.39	1.00
Fatigue	6(18.8)	0(0.0)	9(27.3)	0(0.0)	0.42	-
Fever	4(12.5)	0(0.0)	5(15.2)	0(0.0)	0.76	-
Headache	1(3.1)	0(0.0)	5(15.2)	0(0.0)	0.09	-
proteinuria	0(0.0)	0(0.0)	1(3.0)	1(3.0)	0.32	1.00
hypertension	2(6.3)	0(0.0)	5(15.2)	0(0.0)	0.25	-

Most notably, the incidence of adverse events related to apatinib: hypertension and proteinuria were 15.2% and 3% respectively. No treatment-related nonhematologic grade 4 adverse events or treatment-related deaths were observed. Dose modification (reduction or interruption temporarily) was most commonly due to thrombocytopenia in the vinorelbine group (3.1%) and to leukopenia, granulocytopenia in the apatinib plus vinorelbine group (15.2%). Adverse events resulting in discontinuation of the drug occurred in 1(9%) of 33 patients who received apatinib plus vinorelbine group.

## Discussion

This randomized, open-label, phase 2 trial is the first study to uncover the efficacy and safety of adding apatinib to chemotherapy in patients with advanced TNBC with failed first/second-line treatment. In line with our expectations, we demonstrated that apatinib plus vinorelbine revealed a longer PFS compare with those who received vinorelbine monotherapy (3.9 months vs. 2.0 months; *P* = 0.026). Furthermore, subgroup analysis showed that the addition of apatinib resulted in apparently lower the risk of disease progression among clinically relevant factors including age, menopausal status, number of metastases, previous treatment, visceral metastasis. This is statistically significant but be honest that it is clinically not as significant but how these results could inform future trials. Besides, it must be confessed, added toxicity for a 1.9 month benefit may not be worth it for some patients. Therefore, as a clinician, this regimen should also be selected after balancing radiotherapy and toxicity.

The secondary end point of OS indicated a tendency toward beneficial to survival in the patients with apatinib plus vinorelbine compared to vinorelbine despite statistically insignificant (11.5 months vs. 9.9 months; *P* = 0.985), which could be attribute to the insufficient follow-up time, the end point of death in many patients did not occur at the cut-off time point. Thus, further subgroup analysis among clinically relevant factors in patients will be launched after the overall survival data are mature. Although no significant difference in ORR was observed between apatinib plus vinorelbine treatment and vinorelbine monotherapy, but apatinib plus vinorelbine showed superior over vinorelbine with 9.1% vs. 6.3%, respectively. It is important to note that one (3.0%) of 33 patients in apatinib plus vinorelbine group achieved a CR and two (6.1%) achieved a partial response.

The combination of apatinib plus vinorelbine was considered reasonably well tolerated. Most frequent hematologic grade 3–4 adverse events were leukopenia and granulocytopenia, which was clinically controllable, and rapidly alleviate after dose modification. Since the dose escalation of apatinib (Initial dose: 250 mg; if tolerable, the second cycle: 500 mg), the common toxicity of apatinib: hand–foot syndrome, hypertension and proteinuria were mainly grade 1–2, only one patient developed grade 3 foot syndrome and proteinuria and recovery through dose reduction. Thus, apatinib related adverse events were generally administrable after appropriate clinically intervention. Furthermore, the design of 5-day continuous administration and 2-day rest brought benefits to the recovery of adverse effects of apatinib, thus increasing the tolerance of the drug.

Apatinib as an orally administered antiangiogenic drug, can make the existing tumor vessels degenerate, block the transport of oxygen and other nutrients for tumor growth and inhibit tumor neovascularization, thereby repress metastasis^31^. Importantly, apatinib can normalize the surviving tumor vessels, improve the delivery of chemotherapy drugs through reducing the pressure between tumor tissues, and enhance the efficacy of chemotherapy^32^. Recently, the efficacy of apatinib in advanced TNBC with failure of chemoradiotherapy has been confirmed in several studies^33–35^. In a retrospective study, combination of apatinib and capecitabine achieved a better PFS and tolerable toxicity compare with capecitabine monotherapy, which may regard as one of the options of the third-line treatment for advanced TNBC^36^. In previous NCT 01176669 trial, we evaluated the optimum dose level for the efficacy and safety of apatinib monotherapy in patients with metastatic TNBC. The results indicated that the lower daily dose of apatinib 500 mg/day is active in pretreated metastatic TNBC with perspective CRR, clinical benefit rate and PFS. NAN trail is an extension and replenishment to our previous NCT 01176669 trial, which fully utilized the interval of vascular normalization that may be brought by the administration of apatinib before chemotherapy, so as to maximize the efficacy of vinorelbine. Furthermore, we will further establish a subgroup of metronomic regimen chemotherapy to explore the effect of apatinib combined metronomic regimen on relieving toxicity on the basis of ensuring the curative effect.

Cytotoxic chemotherapy remains the chief treatment for metastatic TNBC as currently there are no endocrine or specific targeted regimes available^37^. Anthracyclines and taxanes are frequently used in the first-line or second-line treatment, the choice of drugs for back-line treatment has encountered a bottleneck^38^. A considerable number of patients have received capecitabine in clinical trials during first few lines^39–41^. Besides, the efficacy of xeloda is uncertain in patients who have relapsed after participating in clinical trials. Therefore, capecitabine was difficult to be chose as a chemotherapy drug in patients with advanced TNBC with failed first/second-line treatment. While, vinorelbine is a commonly used drug for posterior line rescue treatment with response rates of 36–50%^42^. Vinorelbine, a semisynthetic alkaloid from primula oblongata, is a cell cycle-specific drug, which can stop cell division in the metaphase of mitosis by interfering with the polymerization of tubulin^43^. Multiple clinical trials have explored the efficacy of vinorelbine-based later-line setting in metastatic TNBC^44–49^. Our previously prospective phase II trial also indicated that biweekly vinorelbine and oxaliplatin regimen is effective and well-tolerated as second- or third-line treatment for patients with metastatic TNBC^28^. In NAN study, the dose of vinorelbine combined with apatinib can refer to a phase I/II study of vinorelbine combined with sorafenib^50^. Besides, the side effects of apatinib need time to recover in order to increase drug tolerance, so compare with navelbine monotherapy arm, we conducted the dose reduction of navelbine.

Multiple targeted therapies have achieved drastic improvements in the treatment of metastatic TNBC. Recently clinical studies showed that adding programmed death receptor 1 (PD-1) or its ligand (PD-L1) blockade to chemotherapy significantly improved PFS in PD-L1 positive mTNBC patients^51–53^. PARP inhibitors -Olaparib and talazoparib are approved as a standard of care for the treatment of metastatic TNBC harboring a germline BRCA mutations^54–56^. Furthermore, antibody-drug conjugates (ADC) drugs including sacituzumab govitecan demonstrated high activity in pretreated mTNBC in a randomized phase III trial versus single-agent chemotherapy^57,58^.

Unfortunately, no approval has been given for failed multiple lines treatment on checkpoint inhibitors for TNBC in China. However, the clinical trials of PD1 in the treatment of metastatic breast cancer have been carried out, and we believe that data support will be available in the near future^59–61^. In view of the literature reports that there may be synergy between PD1 and small molecule antiangiogenic drugs^62^. Besides, the efficacy of vinorelbine combine with immunotherapy or PARP inhibitors in metastatic TNBC is not fully understood. Therefore, the optimal setting for combining apatinib plus vinorelbine therapy with multiple targeted therapies in patients with advanced TNBC is worth of further exploration.

In agreement with the above data, several limitations must be taken into account. The main limitation is considered to be the insufficient follow‐up time. On the basis of the OS Kaplan‐Meier curves, there remains more than 25% of the patients survived at the end of the follow‐up; the shortest follow-up time was only about 2 months. thus, the outcomes seem less rigorous. Moreover, owing to one patient withdrew consent without receiving treatment, the uneven distribution of patients’ number comes up with a relative basis outcome. Furthermore, in an effort to strengthen and extend above findings, the need for detecting VEGFR tissue biomarker with more survival prognosis outcomes should be launched to further confirm prognosis effectiveness of apatinib plus vinorelbine regime.

In conclusion, our study has elucidated that among patients with advanced TNBC with failed first/second-line treatment, adding apatinib to vinorelbine shows a promising benefit in PFS compared to vinorelbine monotherapy, with an excellent toxicity profile. Trails with a large sample sets and longer follow-up period warranting further exploration.

## Method

### Patients

NAN was a prospective, open label, single-center randomized phase II trial performed in Fudan University Shanghai Cancer Center. Eligible patients were female patients aged 18–70 years, with histologically confirmed recurrent (unresectable) or metastatic TNBC (defined as, ER/PR stain of <1% positive tumor cells with nuclear staining on immunohistochemistry [IHC] and negative HER2 status, defined as 0 or 1+ intensity on IHC, Patients with IHC 2+ were selected to have a fluorescent in situ hybridization test for HER2 gene amplification and the result is negative). Patients were required to have at least one measurable extracranial lesion according to the Response Evaluation Criteria in Solid Tumors (RECIST) version 1.1, an Eastern Cooperative Oncology Group (ECOG) performance status of 0–1 and received maximum of two prior chemotherapy regimens and met the following definitions of treatment failure: progression during the first-line or second-line treatment, or the interval between the follow-up disease progression and the last treatment <3 months. Previous radiotherapy within 4 weeks before enrollment was not permitted. Adequate hematologic (Hb ≥ 90 g/L, No blood transfusion within 14 days; ANC ≥ 1.5 × 109/L; PLT ≥ 75 × 109/L), hepatic (TBIL ≤ 1. 5 × ULN (Upper limit of normal value); ALT and AST ≤ 3 × ULN), and renal (Cr ≤ 1 × ULN) function and a life expectancy of at least 3 months were also required.

Patients with a history of treatment with the vinorelbine, or a history of treatment with the VEGFR tyrosine kinase inhibitors except bevacizumab, any factors interfering with oral medication, a history of psychotropic drug abuse and inability to abstain or mental disorders, any previous clinical trials within 4 weeks before starting NAN trail and women who were pregnant or lactating were excluded. Patients with brain metastases were also excluded unless they were asymptomatic for at least 8 weeks, who do not need glucocorticoid and mannitol to reduce intracranial pressure, and at least one measurable lesion other than brain metastasis, and more than 4 weeks between brain radiotherapy and the last radiotherapy.

Additional exclusion criteria were severe cardiopulmonary, hepatorenal dysfunction, severe or uncontrolled infection, unhealed wound, traumatic bone fracture, hypertension and uncontrolled by antihypertensive drugs, grade >1 myocardial ischemia or myocardial infarction, grade ≥1 arrhythmia (QT prolongation ≥440 ms) or cardiac dysfunction; coagulation disorders, evidence of bleeding diathesis or gastrointestinal bleeding tendency; arteriovenous thrombotic events within 6 months; history of other malignancies within 5 years except basal cell carcinoma of skin and carcinoma in situ of uterine cervix.

The relevant institutional review board or ethics committee of Fudan University Shanghai Cancer Center approved the study, which was conducted in accordance with the Declaration of Helsinki. All patients provided written informed consent.

### Study design and treatment

The NAN trial was an open-label, randomized, phase 2 trial evaluating the safety and efficacy of adding apatinib to chemotherapy in patients with advanced TNBC with failed first/second-line treatment in the metastatic setting. A total of 66 patients were randomly assigned, in a 1:1 ratio, to receive vinorelbine or vinorelbine plus apatinib. Stratification was by visceral metastasis (yes vs. no) and previous treatment (first-line vs. second-line). Vinorelbine monotherapy was administered as a once-per-week intravenous infusion of 25 mg/m^2^ on days 1, 8, and 15 of each 28-day treatment cycle. In vinorelbine plus apatinib group, vinorelbine in administered intravenously at a dose of 20 mg/m^2^ on day 7, 14, and 21 of each 28-day treatment cycle. Apatinib 250 mg was administered orally once daily at a fixed time each day about 30 min after a meal, with treatment on days 1 to 5 of weeks 1, 2, and 3 within each 28-day cycle (if tolerable, the second cycle started with 500 mg per day).

Dose modifications were regulated by protocol-specified toxicity criteria. Tumor assessment of evaluable lesions was performed by computed tomography scanning or magnetic resonance imaging at baseline (4 weeks before treatment), every two cycles (every 6 weeks ± 3 days) during treatment, and ECOG was performed at the same time. According to the RECIST 1.1 criteria^63^, patients with CR, PR and SD continued treatments until disease progression, development of unacceptable toxicity, or withdrawal of consent.

The primary endpoint was progression-free survival (PFS), which was defined as the time from randomization to objective tumor progression (according to RECIST 1.1) or death from any cause, whichever occurred first. Secondary end points included overall survival (OS), overall response rate (ORR) and safety. OS was defined as the time from randomization to last follow up or death from any cause. ORR was defined as the proportion of patients whose best objective response was confirmed complete or partial response before disease progression. Safety and adverse events including Hematological and nonhematological toxicity were graded and assessed with the use of the National Cancer Institute Common Terminology Criteria for Adverse Events (NCI CTCAE version 4.0).

### Statistical analysis

According to the available data on phase III trials enrolling TNBC patients, the PFS with vinorelbine plus apatinib would have been increased from 2.5 months of vinorelbine monotherapy to 5.5 months (24 months enrollment duration, 12 months follow-up duration after enrollment), with 80% power at a 5% significance level. A sample of 66 evaluable patients were to be enrolled assuming <10% patient discontinuation rate.

Statistical analyses were performed using SPSS 23.0 for Windows (IBM, Armonk, NY, USA). The means were calculated for age variable, and percentages were calculated for clinicopathological variables. Survival end points were constructed by the Kaplan‐Meier method, and the difference was detected by log‐rank test. Subgroup analyses were performed using a stratified Cox proportional-hazards model with two-sided 95% confidence intervals. Efficacy data were analyzed on an intention-to-treat basis. Categorical variables analysis was performed using the Pearson test or the Fisher exact test. Safety was assessed in all patients who received at least one dose of the trial treatment. The grade of adverse event during trial treatment was reported. All statistical tests were two‐sided., differences were considered statistically significant if *P* < 0.05.

This study is registered with ClinicalTrials.gov Identifier: NCT03254654, August 17, 2017. Disclosures provided by the authors and data availability statement (if applicable) are available upon request by contact with the corresponding author.

## Data Availability

The datasets generated and analyzed during the current study are available from the corresponding author on reasonable request.
